# Supplementary material for: Why women do not use contraceptives: Exploring the role of male out-migration
Source: PLoS One. 2021 Mar 30;16(3):e0249177. doi: 10.1371/journal.pone.0249177 (PMC8009410; doi:10.1371/journal.pone.0249177)
Supplement: Attachment — Submitted filename: Response.docx [file pone.0249177.s002.docx]

**Manuscript title:** Why women do not use contraceptives: Exploring the role of male out-migration

**Manuscript ID:** PONE-D-20-13937R1

Dear Dr. Zimmerman,

Academic Editor

PLOS ONE

Thank you for giving us an opportunity to address the additional suggestions from the reviewer 3. We have made the necessary changes to the manuscript. Following is our response to the reviewers' comments, as required.

**Response to the reviewer # 3:**

***Overall:***

Since PLOS One does not copyedit accepted manuscripts, the authors need to copyedit the manuscript carefully. This is particularly applicable to text that was added in this revision -- there are some missing articles ("a", "the", etc) and typos.

**Response:** Thank you. We have done a thorough editing of the manuscript.

***Study design:***

1. The justification for using FGDs still seems unclear, as divergent views can also emerge by interviewing people individually (in fact, IDIs may be more likely to elicit divergent views on sensitive topics because people are more likely to speak freely). Do the authors mean to say that they wanted to capture broader community perceptions and norms on contraceptive use rather than people's individual contraceptive practices?

**Response:** Yes, we wanted to capture the broader community perceptions.

2. Can the authors please clarify the following statement: "The other objective of conducting FGD was to inform the quantitative survey in developing study tool tuned for study context." What quantitative survey? How would FGD findings be preferable to in-depth interview findings in informing development of a quantitative tool?

**Response:** Thank you for highlighting this. FGDs were not only informed in framing the right questions for migration context but also guided the analysis of quantitative survey data. In terms of survey tool development, it helped in framing the questions in local language and picking the right terms to be used in question framing and response categories. In case of In-depth interviews, terms prevalent at community level may not have emerged so clearly.

The quantitative survey was followed by the qualitative phase where the objective was to examine if the contraceptive use behaviours of women in migration affected areas were linked to migration of women’s husband. A paper based on quant data has been published and can be accessed here: [https://bmcpublichealth.biomedcentral.com/articles/10.1186/s12889-020-09906-9](https://nam02.safelinks.protection.outlook.com/?url=https%3A%2F%2Fbmcpublichealth.biomedcentral.com%2Farticles%2F10.1186%2Fs12889-020-09906-9&data=04%7C01%7Csmukherjee%40popcouncil.org%7Cbc3f77cce64a40106d2908d8ccb3dae7%7Cda295b63c8454fa2990b1ab42d54f2ca%7C1%7C0%7C637484420137666010%7CUnknown%7CTWFpbGZsb3d8eyJWIjoiMC4wLjAwMDAiLCJQIjoiV2luMzIiLCJBTiI6Ik1haWwiLCJXVCI6Mn0%3D%7C1000&sdata=GNQO3F5l7a7rdRPPoYzEFw4rrPUPvMEOYTaOJFtIjUc%3D&reserved=0)

3. In their response to the reviewers, the authors explained why they selected two high male out-migration and two low male out-migration blocks in each district, and indicated that this stratification was dropped in the analysis phase as no major differences in themes emerged. However, this explanation was not added to the manuscript text. Please add this explanation.

**Response:** Thank you for pointing this out. We have included a sentence to this effect in the revised manuscript.

4. The following sentence is unclear, please revise: "At the end of the study, while from each study group, one FGD was conducted in each block, for men in one block, an additional FGD was conducted to capture the diverging views of population." Are the authors saying that the FGDs didn't elicit many divergent views, so they conducted one additional FGD at the end of the study to try to elicit divergent views?

**Response:** Thank you for point this out. In one of the blocks FGDs for migrant and non-migrant men were collected separately. We meant that the view of these groups was very different. In the revised manuscript, we have clarified this.

***Results:***
1. I am a bit confused about why "poor couple communication on FP" is categorized as a characteristic of the migration environment. This seems directly related to the husband's outmigration, since it means that couples talk infrequently and need to reserve those conversations for essential topics. Perhaps this could be recategorized?

**Response:** Poor couple of communication on FP was prevalent across all couples residing in the migration affected areas irrespective of whether women had migrant and resident husband. Couples generally refrained from discussing about contraceptives amongst themselves. This was emerged from discussion with men and ASHAs as well. We have made some revision to the text to bring more clarity around this.

2. "Fear of sickness due to side effects" -- suggest to rename this simply "Fear of side effects", and move the content on side effects up to this section (see next comment).

**Response:** We have changed the “Fear of sickness due to side effects” to “Fear of side effects” in the revised manuscript.

3. The first paragraph under "myths and misperceptions" describes legitimate side effects that can be experienced with contraceptive method use, as well as misperceptions (i.e. contraceptive methods cause cancer). I would suggest to move the content about fear of side effects to the section above "Fear of sickness due to side effects", and reserve this section for true myths/misperceptions.
**Response:** Thank you again for bringing this to our attention. As suggested, in the revised manuscript we have moved the content about fear of side effects to the section "Fear of side effects” and revised the text on myths and misperceptions section to make it more relevant.

***Discussion:***
1. Page 15: "Whereas, at the system level, low ASHA outreach on FP, need to prove fertility, preference of male child, myths and misconception about contraception, poor couple communication on FP were listed as deterrents to contraceptive use." -- The term "system level" seems appropriate when talking about ASHA outreach and lack of accurate communication on contraception, but the other factors don't quite fit under this term. Suggest to use a more comprehensive term like "system-level and community/cultural factors".

**Response:** Thanks for highlighting this. We have made the suggested change in the revised manuscript.

2. Limitations: I recommend that the authors acknowledge the potential drawbacks of using FGDs to discuss sensitive topics like contraception -- namely that people may not feel comfortable to openly express their views in a large group, particularly if their opinion differs from others / the loudest voices in the group.

**Response:** We have included the limitation with FGDs in the revised manuscript.

We again thank the reviewers and you for giving us this opportunity and will be happy to provide further clarification if required.

With warm regards

Bidhu

[bbmahaptra@gmail.com](mailto:bbmahaptra@gmail.com)
